# Inositol 1,4,5-trisphosphate receptors in cardiomyocyte physiology and disease

**DOI:** 10.1098/rstb.2021.0319

**Published:** 2022-11-21

**Authors:** Kateryna Demydenko, Samaneh Ekhteraei-Tousi, H. Llewelyn Roderick

**Affiliations:** Laboratory of Experimental Cardiology, Department of Cardiovascular Sciences, KU Leuven, Leuven, Belgium

**Keywords:** calcium signalling, cardiomyocyte, excitation contraction coupling, InsP_3_R, cardiac hypertrophy, calcium microdomains

## Abstract

The contraction of cardiac muscle underlying the pumping action of the heart is mediated by the process of excitation-contraction coupling (ECC). While triggered by Ca^2+^ entry across the sarcolemma during the action potential, it is the release of Ca^2+^ from the sarcoplasmic reticulum (SR) intracellular Ca^2+^ store via ryanodine receptors (RyRs) that plays the major role in induction of contraction. Ca^2+^ also acts as a key intracellular messenger regulating transcription underlying hypertrophic growth. Although Ca^2+^ release via RyRs is by far the greatest contributor to the generation of Ca^2+^ transients in the cardiomyocyte, Ca^2+^ is also released from the SR via inositol 1,4,5-trisphosphate (InsP_3_) receptors (InsP_3_Rs). This InsP_3_-induced Ca^2+^ release modifies Ca^2+^ transients during ECC, participates in directing Ca^2+^ to the mitochondria, and stimulates the transcription of genes underlying hypertrophic growth. Central to these specific actions of InsP_3_Rs is their localization to responsible signalling microdomains, the dyad, the SR-mitochondrial interface and the nucleus. In this review, the various roles of InsP_3_R in cardiac (patho)physiology and the mechanisms by which InsP_3_ signalling selectively influences the different cardiomyocyte cell processes in which it is involved will be presented.

This article is part of the theme issue ‘The cardiomyocyte: new revelations on the interplay between architecture and function in growth, health, and disease’.

## 1. Ca^2+^ and the heart

Ca^2+^ is a pleiotropic intracellular messenger controlling key aspects of cardiac biology [1]. Of particular importance is its role in the physiology of the cardiomyocyte, where global increases in its intracellular concentration couple electrical depolarization of the sarcolemma during excitation-contraction coupling (ECC) with contraction [2,3]. Supporting this role in ECC and other cell processes, Ca^2+^ is taken up into the mitochondria to stimulate metabolism, generate ATP required for contraction and mediate Ca^2+^ clearance from the cytosol during relaxation. Ca^2+^ transients underlying contraction are acutely tuned to the cardiovascular needs of the organism, being augmented in amplitude and kinetics under periods of increased sympathetic drive, such as during the fight-or-flight response. Further, and consistent with this role in coupling cardiac output with haemodynamic requirements, via stimulation of gene expression, alterations in Ca^2+^ induce hypertrophic growth of the heart required for sustained increases in demand. Such hypertrophic growth occurs during developmental growth, pregnancy and during disease processes such as in response to cardiac damage following an infarct. When dysregulated, Ca^2+^ is involved in cardiomyocyte cell death processes and importantly in cardiac pathologies including in mediating arrhythmic activity and in the reduction in cardiac output during heart failure. The diversity of these functions of Ca^2+^ in the cardiomyocyte suggests the requirement for complex mechanisms for Ca^2+^ signal modulation to ensure discrete encoding of its involved cell processes.

## 2. Inositol 1,4,5-trisphosphate signalling in the heart

**Figure 1.**
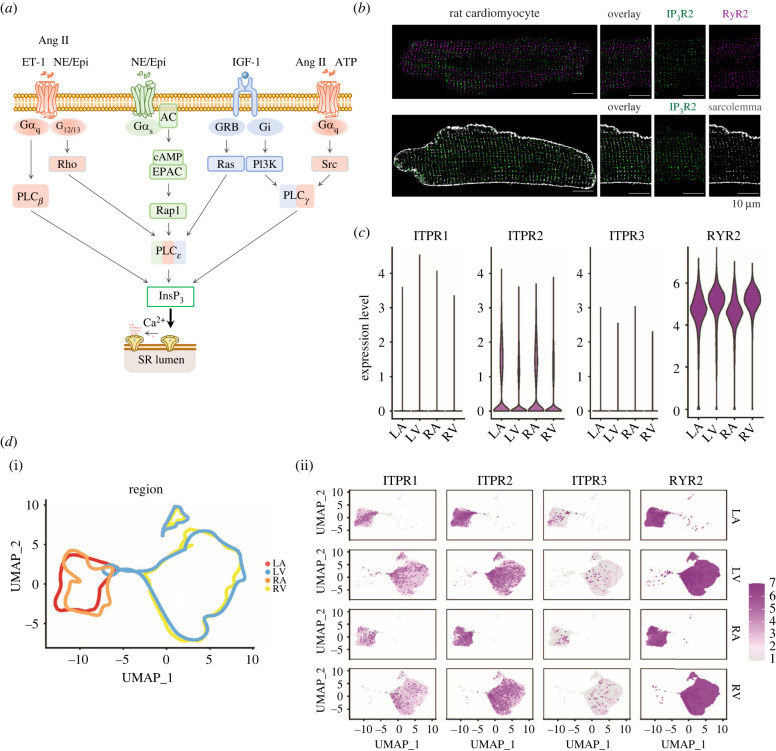
InsP_3_R expression in the heart. (*a*) InsP_3_ is generated by activated phospholipase C (PLC) following the engagement of G-protein coupled receptors (GPCRs) liganded by either angiotensin II (Ang II; angiotensin receptor, AT_1_), endothelin-1 (ET-1; endothelin receptor, ET receptors), adenosine triphosphate (ATP; purinergic receptors, P2Y), the catecholamines (CA; *α* and β-adrenoreceptors, α-AR and β-AR) epinephrine (Epi) and norepinephrine (NE) and insulin-like growth factor 1 (IGF-1; IGF-1 receptor, IGF-1R). After diffusion into the cytosol or the nucleus, InsP_3_ binds to each subunit within the InsP_3_R tetramer leading to channel opening and release of Ca^2+^ from intracellular Ca^2+^ storage sites. (*b*) InsP_3_R2 localization relative to RyR2 (top) and t-tubules (bottom). InsP_3_Rs are stained in green, RyRs are stained in purple and the t-tubules (Caveolin (Cav3)/NCX) are in grey. A 4× zoom of the white square is shown. (*c*) Log normalized expression of the genes encoding the three inositol 1,4,5-trisphosphate receptors (*ITPR1-3*) and the gene encoding the type 2 RyR (*RYR2*) in the cardiomyocyte single nucleus RNA-Seq from each of the four heart chambers. (*d*) (i) Uniform Manifold Approximation and Projection for dimension reduction (UMAP) embedding of the cardiomyocytes from the four heart chambers including 14 772 nuclei from left atrium (LA), 41 699 nuclei from left ventricle (LV), 8711 nuclei from right atrium (RA) and 30 915 nuclei from right ventricle (RV). These data are from a recent publication by Litviňuková *et al*. [9], which included transcriptomes of cardiomyocyte nuclei harvested from 14 individuals from two main sources (Harvard Medical School and Wellcome Sanger Institute) and that were processed using Chromium Controller (10× Genomics). Dots representing the nuclei within the UMAP are removed and lines encompassing the nuclei per heart chamber are retained to illustrate the distribution of the nuclei from each heart region relative to other regions in the UMAP. (ii) Distribution of the ITPR /RyR2 expressing cardiomyocytes across the separated heart regions (colour intensity is binned according to the maximum log normalized value of RyR2 expression).

During ECC, the cell-wide increase in [Ca^2+^]_i_ required for induction of contraction is generated by the Ca^2+^-dependent activation of ryanodine receptors (RyR) Ca^2+^ release channels located on the sarcoplasmic reticulum (SR) intracellular Ca^2+^ store by Ca^2+^ entering the cell through sarcolemmal L-type voltage-gated Ca^2+^ channels (LTCC) [3,4]. During this process of Ca^2+^-induced Ca^2+^ release (CICR), Ca^2+^ release from the SR dominates over Ca^2+^ entry by approximately 10 : 1 and is thus primarily responsible for cardiomyocyte contraction. In addition to RyRs, cardiomyocytes also express inositol 1,4,5-trisphosphate receptors (InsP_3_R) Ca^2+^ release channels that are also located on the SR Ca^2+^ store. While RyRs play a central role in the generation of Ca^2+^ signals underlying ECC, the contribution of Ca^2+^ release from InsP_3_Rs to cardiomyocyte physiology is not so clear. In contrast to RyRs, which are activated and inhibited by Ca^2+^ [5], InsP_3_Rs require both InsP_3_ and Ca^2+^ for full activation [6–8]. As in other cell types, InsP_3_ is generated in cardiomyocytes via phospholipase C (PLC)-mediated hydrolysis of phosphatidyl inositol 4,5-bisphosphate (PtdIns4,5P_2_). The mechanism of PLC activation is dependent on the isoform involved. PLC*β* isoforms are activated by G*α*_q_ following engagement of G-protein coupled receptors (GPCR), PLC*γ* by recruitment to receptor tyrosine kinases (RTK) and PLC*ε* by Rho, Ras and Rap pathways activated downstream of GPCR and RTK (figure 1*a*). Cardiomyocytes express a number of GPCRs that respond to an array of locally produced or circulating neurohormones and peptides including *α*1-adrenoceptors, angiotensin (AT), endothelin and purinergic receptors liganded by catecholamines (norepeinephrine and epinephrine or synthetic *α*1-AR ligand phenylephrine), angiotensin II (Ang II), endothelin-1 (ET-1) and ATP, respectively [10]. These ligands and their receptors are engaged during both physiology and pathophysiology where they play roles in stress adaptation and tissue remodeling. Despite expression of these receptors in cardiomyocytes, levels of InsP_3_ produced following their engagement is by comparison with other cell types, relatively low [11]. The effects of receptor engagement may also be long lasting owing to continued activity subsequent to receptor endocytosis [12,13]. Growth factor receptors such as the insulin-like growth factor 1 receptor (IGF1R) (from the family of tyrosine kinase receptors) via activation of GPCRs docked to a pertussis toxin-sensitive heterotetrameric G_i_ protein, also stimulate the generation of InsP_3_ upon their engagement [14]. Nuclear anchored PLC*ε* activated downstream of Ras-MAPK and cAMP signalling produces InsP_3_ locally [15]. Significantly, PLC*ε* may be activated by G_12-13_-dependent Rho, cAMP-Epac and Ras pathways engaged downstream of ET_A_ receptors, β-adrenoceptors and IGF1Rs, respectively.

Expression of all three InsP_3_R isoforms is reported in cardiomyocytes. The type 2 InsP_3_R (InsP_3_R2) is however most prevalent, albeit at varying levels with an approximately sixfold greater abundance in atria than in the ventricles [16,17]. The type 1 InsP_3_R (InsP_3_R1) is most abundant in the fetal heart, although both type 1 and 3 InsP_3_Rs have also been detected in the adult. In line with these previous reports, our interrogation of the cardiomyocyte compartment (identified by their transcriptomes) in a published dataset of single nucleus RNA-sequencing data from different regions of the human heart also show predominance of InsP_3_R2 in cardiomyocytes from all heart regions (figure 1*c–d*) [9]. Expression of InsP_3_R1 is also detected in the heart, especially in the atria albeit at a substantially lower level than InsP_3_R2, while InsP_3_R3 is almost absent. The biophysical properties of InsP_3_R2 make it most appropriate for its function in cardiomyocytes. It exhibits the greatest InsP_3_ sensitivity of the three isoforms (InsP_3_R2 > InsP_3_R1 > InsP_3_R3) and thus can be activated by the low InsP_3_ concentrations (10–30 nM) produced following neurohormonal stimulation in cardiomyocytes [11,18]. In contrast to the clear role of RyRs in generating Ca^2+^ signals during ECC, the function of InsP_3_Rs in cardiomyocytes is not as defined and is less consistent between studies. This is perhaps not surprising considering the lower level of InsP_3_R expression and the less obvious contribution of Ca^2+^ release via these receptors to Ca^2+^ handling [16,19]. These observations raise the question whether InsP_3_Rs are required for normal cardiomyocyte function in the adult or whether their existence is simply a vestige of an earlier developmental stage. Indeed, Ca^2+^ release via InsP_3_Rs underlies the first heart beat [20–22] and InsP_3_Rs are more highly expressed during early development than in the adult [23]. Moreover, InsP_3_Rs are required for compaction of the myocardium and valve formation during development [24,25]. Further, the lack of an overt heart phenotype in InsP_3_R2 knockout adult mice would suggest that InsP_3_Rs are not required for the normal physiological function of cardiomyocytes during adulthood [26]. By contrast, in contexts of greater InsP_3_R2 expression such as in the atria or in the diseased ventricle, where InsP_3_R expression is heightened, a clearer picture of InsP_3_R biology is emerging. For example, InsP_3_R2 knockout protects against ET-1-induced arrhythmias in atrial cardiomyocytes and improves cardiac function in ischemic heart disease respectively [26,27]. Supporting the additional role of InsP_3_R2 in regulation of cardiac hypertrophy, transgenic mice engineered to selectively overexpress InsP_3_R2 in cardiomyocytes develop mild hypertrophy and exhibit increased arrhythmias [28,29]. Despite some reports showing a minor or no involvement of InsP_3_Rs in the actions of neurohormonal stimuli, the weight of evidence would indicate that InsP_3_Rs contribute to intracellular signalling evoked by neurohormonal stimulation of cardiomyocytes [26,30–32]. Moreover, through localization to subcellular compartments and responsiveness to InsP_3_ generated in cardiomyocytes, InsP_3_-induced Ca^2+^ release (IICR) is now established as a unique signal involved in the regulation of diverse cell functions including ECC, metabolism and gene expression. How InsP_3_ signalling contributes to, and selectively influences various cell processes in cardiomyocytes is discussed in the following sections.

## 3. Inositol 1,4,5-trisphosphate in excitation-contraction coupling

By mediating Ca^2+^ release from the SR, a role for InsP_3_Rs in ECC can be explained [16]. The contribution of Ca^2+^ release via this mechanism to ECC is however inconsistent between studies, ranging from no effect to altered dynamics of Ca^2+^ transients and increased propensity of spontaneous Ca^2+^ release events [28,31,33–35]. These divergent effects may be ascribed to differences in expression, intracellular localization and activity of InsP_3_Rs associated with the heart region, animal model and developmental stage. Indeed, in immature [20–22], atrial [32,35–38] and diseased adult ventricular cardiomyocytes [31,39,40] where InsP_3_R expression is greatest, an influence of InsP_3_Rs on ECC is consistently observed. Early studies revealed a much greater InsP_3_R abundance and hence an effect of InsP_3_-generating stimuli on the contractility of atrial preparations than upon ventricular counterparts [16,36]. To influence ECC, the location of InsP_3_Rs is important. In both atrial and ventricular cardiomyocytes, InsP_3_Rs substantially co-locate with junctional RyRs [33,41]. InsP_3_Rs are thus observed in a striated pattern along the Z-lines coinciding with RyRs. In ventricular cardiomyocytes, owing to the presence of t-tubules (TTs), InsP_3_Rs are thus located in specialized structures termed dyads [33,42,43]. In atrial cells, while co-located with RyRs along the Z-lines, InsP_3_Rs are also enriched at sub-sarcolemmal regions, where they co-localise with RyRs [16,41,44].

### (a) Atria

**Figure 2.**
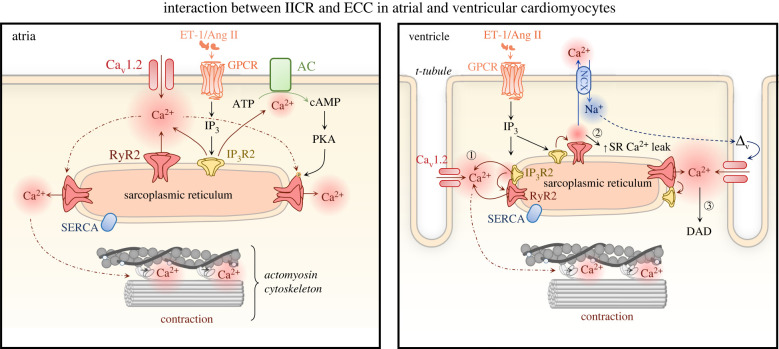
Mechanisms of InsP_3_-mediated regulation of ECC in atrial and ventricular cardiomyocytes. Atria: GPCRs activated by ET-1 or Ang II produce InsP_3_ that stimulates Ca^2+^ release via InsP_3_ receptors type 1 or 2 (InsP_3_R1/2). This InsP_3_ mediated Ca^2+^ release in turn acts either via priming of proximal RyRs for Ca^2+^ release or via activation of Ca^2+^-sensitive adenylyl cyclases (AC1 or AC8) and activation of PKA by cAMP, which then phosphorylates RyRs, modulates Ca^2+^ transients and hence strength of contraction. Ventricle: Ca^2+^ release via InsP_3_Rs facilitates RyR opening and enhances their recruitment during ECC (1). However, the enhanced activity of RyRs leads also to enhanced SR Ca^2+^ leak (2), which reduces the Ca^2+^ load in the SR and can lead to activation of NCX. If the SR Ca^2+^ leak is of sufficient amplitude, via NCX, it can trigger substantial Na^+^ influx into the cell leading to membrane depolarization manifest as a delayed after-depolarisation (DAD) and potentially AP generation (3). AC, adenylyl cyclase; Ang II, angiotensin II; ATP, adenosine-5′-triphosphate; cAMP, cyclic adenosine monophosphate, Ca_v_1.2, *α*1C, subunit of voltage-gated L-type calcium channel; DAD, delayed after-depolarizations; ET-1, endothelin 1; GPCR, G protein-coupled receptor; IP_3_, inositol 1,4,5-trisphosphate; IP_3_R1/2, inositol trisphosphate receptor type 1/2; NCX, sodium-calcium exchanger; PKA, protein kinase A; RyR2, ryanodine receptor type 2; SERCA, sarco-endoplasmic reticulum Ca^2+^-ATPase; SR, sarcoplasmic reticulum. (Online version in colour.)

Both type 1 and 2, InsP_3_Rs are expressed in atrial cardiomyocytes [16,45,46]. In these cells, irrespective of species analysed, InsP_3_R activation results in increased Ca^2+^ mobilization from the SR [36–38,47]. Specifically, InsP_3_R activation leads to an increased amplitude of Ca^2+^ transients in the sub-sarcolemmal region and in regions distal to the periphery, thereby augmenting the magnitude of cell-wide Ca^2+^ transient [41,48,49] (figure 2, atria). In addition to effects on the electrically-evoked Ca^2+^ transient during ECC, activation of InsP_3_ signalling contributes to an increase in the incidence of extra-systolic Ca^2+^ elevations and spontaneous contractions in atrial cardiomyocytes exposed to ET-1 and Ang II [36,41,48]. Supporting the involvement of InsP_3_R2 in these pro-arrhythmic effects of GPCR ligands, spontaneous Ca^2+^ elevations are absent in atrial cardiomyocytes from InsP_3_R2 knock-out mice [26]. Underlying and likely contributing to these arrhythmic events induced by GPCR agonists or InsP_3_ is a substantial increase in occurrence of Ca^2+^ sparks [16,36,37,41,48]. As a consequence of this increased Ca^2+^ spark frequency, diastolic Ca^2+^ levels have also been reported in atrial cardiomyocytes under conditions of GPCR or InsP_3_ stimulation. The aforementioned effects are particularly pronounced in atrial cardiomyocytes from hypertrophic hearts, in which InsP_3_R expression is greater [45,46,48,50]. The greater InsP_3_R abundance may have dual consequences however. While initially, increased InsP_3_R activity enhances atrial contractility to augment their capacity to propel blood into the ventricle, the constitutive activation of InsP_3_Rs is deleterious. Specifically, through more frequent spontaneous Ca^2+^ releases and/or greater SR Ca^2+^ leak, constitutive activation of InsP_3_Rs leads to a reduction in SR load with associated suppression of Ca^2+^ transients as well as to an augmentation of inward Na^+^/CA^2+^ exchanger (NCX) current and membrane depolarization, that if of sufficient magnitude can trigger delayed after-depolarization or action potentials (APs) [48]. This increased abundance and activity of InsP_3_Rs in pathology potentially combines with RyRs sensitized by hyperactive Ca^2+^/calmodulin-dependent protein kinase II (CaMKII) and protein kinase A (PKA) to create a perfect storm of dysregulated Ca^2+^ release that generates cell-wide and tissue arrhythmia [51].

Increasing evidence supports the notion that Ca^2+^ release via InsP_3_Rs shapes global Ca^2+^ transients and thereby atrial cardiomyocyte contractility via functional crosstalk with RyRs, whereby SR Ca^2+^ flux via InsP_3_Rs primes proximal RyRs for Ca^2+^ release [36,40]. Support for this mechanism comes from experiments, where IICR is monitored under conditions of RyR inhibition and from the use of advanced imaging methodologies. A recent study from the Egger group imaged facilitation of RyR opening by a preceding Ca^2+^ puff (elementary Ca^2+^ release events through InsP_3_Rs) [40]. Unlike for RyR-mediated Ca^2+^ sparks, the direct detection of events solely arising from InsP_3_Rs is experimentally challenging owing to their smaller magnitude and a lower probability of occurrence. Moreover, conditions under which expression of InsP_3_Rs is elevated maybe required [33,36,40]. The reports of an absence of discrete events in the presence of RyR inhibition but enhancement of RyR-mediated Ca^2+^ sparks has led to the coining of the term ‘eventless Ca^2+^ release via InsP_3_Rs' [37]. This form of Ca^2+^ release from the SR is uncovered under conditions of RyR and NCX inhibition preventing Ca^2+^ extrusion from the cell, thereby allowing Ca^2+^ released via InsP_3_Rs to accumulate in the cytosol [52]. The eventless Ca^2+^ release via InsP_3_Rs is proposed to recruit neighbouring RyR clusters through increased [Ca^2+^]_i_ in their vicinity. Besides functional interactions with RyR, IICR has recently been reported to regulate atrial Ca^2+^ transients through a mechanism involving activation of the Ca^2+^ stimulated adenylyl cyclases, AC1 and AC8 [44] (figure 2, atria). As for coupling to RyRs, IICR engagement of ACs is possible owing to the proximity of the involved proteins, whereby InsP_3_Rs are co-localized with AC8 and are in the vicinity of AC1 in the sub-sarcolemmal region. At this location, IICR activation of AC8 or AC1 and consequent generation of cAMP leads to activation of PKA, which in turn affects Ca^2+^ handling.

### (b) Ventricle

In line with their lower expression in cardiomyocytes of this heart region, the influence of InsP_3_R on ECC in the healthy ventricle is substantially less than in the atria [34,35]. Despite this low expression however, InsP_3_Rs elicit a surprisingly potent effect on ECC [28,31,33]. Probably owing to this lower expression, reported effects on ECC are not consistent (table 1). Subsequent analysis of Ca^2+^ dynamics in cardiomyocytes exposed to InsP_3_, either introduced through cell permeabilization, via a patch pipette or using a cell permeant form of InsP_3_—revealed complex effects on Ca^2+^ handling [33,35,42,48] (figure 2, ventricle). These include increased amplitude of electrically-evoked Ca^2+^ transient, greater propensity of ectopic Ca^2+^ elevations and increased frequency of Ca^2+^ sparks. Notably, the magnitude of the increase in Ca^2+^ transient amplitude elicited by InsP_3_ is not substantial, ranging between a 1.2 and 1.5-fold increase [33,48]. As in atrial cardiomyocytes, G*α*_q_-coupled GPCR engagement by ET-1, Ang II or catecholamines represents the physiological mechanism by which InsP_3_ levels are elevated in ventricular cardiomyocytes. Although these agonists often induce an increase in systolic Ca^2+^ transient amplitude as well as a positive inotropic response, the contribution of InsP_3_ signalling to the action of these GPCRs is often variable or not conclusively established. Indeed, InsP_3_-mediated Ca^2+^ signals are reported to contribute to the inotropic effects of ET-1 in the rabbit [35], but not in the rat [33,34]. Elsewhere, both in human and mouse cardiomyocytes, activation of the GPCR/InsP_3_/InsP_3_R axis causes enhancement of pacing-evoked Ca^2+^ transients and cell contraction [31]. In these aforementioned studies, however, the contribution of InsP_3_Rs to the effects of GPCR activation is not fully established, particularly in humans. Such species discrepancies are likely due to the differences in ET, AT or *α*1-adrenoceptor density and downstream activated signalling pathways. Variability between the effects of agonists may also arise owing to their limited capacity to acutely elevate InsP_3_ levels and thus an effect on Ca^2+^ handling is only observed after prolonged exposure to the GPCR agonist.

**Table 1.** Differential effect of InsP_3_ signalling on cardiomyocyte contractility and Ca^2+^ handling. (Ang II, angiotensin II; ATP, adenosine triphosphate; CaT, Ca^2+^ transient; CMs, cardiomyocytes; ET-1, endothelin-1; IP_3_, inositol 1,4,5-trisphosphate; NRVMS, neonatal rat ventricular cardiomyocytes; PE, phenylephrine; SHR, spontaneously hypertensive rat; SR, sarcoplasmic reticulum; WKY, Wistar-Kyoto strain of rat.)

species	cell type	agonist	observations	reference
rat	NRVMs	PE	↑ frequency of spontaneous CaT in cytosol	[53]
		IP_3_	↑ Ca^2+^ spark frequency in cytosol/nucleus	
			↑ number of Ca^2+^ waves in nucleus	
mouse	ventricular CMs	Ang II	↑ CaT	[54]
mouse	ventricular CMs	ET-1	↑ CaT	[55]
human (healthy and failing)	ventricular CMs		↑ contractility	[31]
			↑ CaT	
			↑ frequency of extra-systolic Ca^2+^ elevations	
		ATP	↑ after-contractions during resting period	
		ET-1	↑ rare spontaneous/sustained Ca^2+^ elevations	
			↓ resting membrane potential	
			↑ duration of the action potential	
			↑ frequency of early after-depolarization	
mouse	ventricular CMs	ET-1	↑ contractility	
		ATP	↑ CaT	
		Ang II	↑ diastolic [Ca^2+^]_i_	
		PE	↑ after-contractions and prolonged contractures	
			↑ extra-systolic and sustained Ca^2+^ elevations	
			↓ resting membrane potential	
			↑ duration of the action potential	
			↑ frequency of early after-depolarization	
rat (WKY and SHR)	ventricular CMs	IP_3_ ester	↑ contractility	[33]
		ET-1	↑ CaT	
			↑ frequency of extra-systolic Ca^2+^ elevations	
			↑ rate of rise of CaT	
			↑ diastolic [Ca^2+^]_i_	
			↑ frequency of Ca^2+^ sparks in the cytosol	
rat	ventricular CMs	ET-1	↑ CaT	[30]
		IP_3_ ester	↑ frequency of extra-systolic Ca^2+^ elevations	
rat	ventricular CMs	ET-1	↓ contractility (2 min post-stimulation)	[34]
			↑ contractility (20 min post-stimulation)	
			↑ rate of contraction (20 min post-stimulation)	
			↑ CaT (amplitude; rate of rise) (20 min post-stimulation)	
rabbit	ventricular CMs	IP_3_	↑ Ca^2+^ leak in the presence of ruthenium red	[52]
rabbit	ventricular CMs	IP_3_	↑ frequency of Ca^2+^ sparks (immediately observed)	[35]
		ET-1	↓ CaT (2 min post-stimulation)	
			↑ CaT (15 min post-stimulation)	
mouse (healthy and failing)	ventricular CMs	Ang II	↑ diastolic [Ca^2+^]_i_	[56]
			↑ CaT	
mouse (IP_3_ overexpression)	ventricular CMs	ET-1	*wild-type:*	[28]
			↑ CaT	
			↑ SR Ca^2+^ load	
			↑ probability of Ca^2+^ wave occurrence	
			*IP_3_-overexpression:*	
			↓ probability of Ca^2+^ wave occurrence sustained SR Ca^2+^ leak	
		IP_3_-salt	*wild-type:*	
			↑ frequency of Ca^2+^ sparks	
			↓ SR Ca^2+^ load	
			*IP_3_-overexpression:*	
			↓ frequency of Ca^2+^ sparks	
			↓ SR Ca^2+^ load	
			no Ca^2+^ puffs were detected	
			unaltered properties of Ca^2+^ sparks	
dog (atrial fibrillation)	atrial CMs	ATP	↑ CaT	[50]
			↑ number of Ca^2+^ transients	
rat	atrial CMs	ET-1	↓ contractility (4 min post-stimulation)	[49]
			↑ contractility (as from 8 min post-stimulation)	
			↓ CaT (4 min post-stimulation)	
		IP_3_ ester	↑ CaT (as from 8 min post-stimulation)	
			↑ frequency of extra-systolic Ca^2+^ elevations	
			↑ frequency of Ca^2+^ sparks	
rabbit	atrial CMs	ET-1	↑ CaT	[32]
			↓ decay *τ* of CaT	
			↑ time to peak of CaT	
dog (atrial fibrillation)	atrial CMs	ET-1	↑ CaT (in nucleus of diseased animals)	[46]
		IP_3_	↑ diastolic [Ca^2+^]_i_	
rat	atrial CMs	IP_3_ ester	↑ CaT	[16]
			↑ frequency of Ca^2+^ sparks	
			↑ frequency of extra-systolic Ca^2+^ elevations	
mouse	atrial CMs	ET-1	↑ CaT	[57]
			↑ diastolic [Ca^2+^]_i_	
			↑ frequency of Ca^2+^ sparks	
			↑ frequency of extra-systolic Ca^2+^ elevations	
cat	atrial CMs	ET-1	↑ CaT (4 min post-stimulation)	[36]
			↑ diastolic [Ca^2+^]_i_	
			↑ frequency of Ca^2+^ sparks (immediately observed)	
			↑ frequency of extra-systolic Ca^2+^ elevations	
	atrial/ventricular CMs	IP_3_	*atrial CMs:*	
			↑ frequency of Ca^2+^ sparks (immediately	
		adenophostin	observed)	
			↑ diastolic [Ca^2+^]_i_	
			↑ diastolic [Ca^2+^]_i_ and frequency of Ca^2+^ puffs in the presence of tetracaine	
			*ventricular CMs:*	
			⇔ frequency or properties of Ca^2+^ sparks	
cat	atrial CMs	IP_3_	↑ diastolic [Ca^2+^]_i_	[58]
		adenophostin	↑ frequency of Ca^2+^ sparks	
cat	atrial CMs	PE	↑ L-type Ca^2+^ current	[59]
rabbit	atrial CMs	caged-IP_3_	↑ CaT	[38]
		IP_3_ ester	↑ diastolic [Ca^2+^]_i_	
			↑ frequency of Ca^2+^ puffs in the presence of tetracaine	
rabbit (healthy and failing)	atrial/ventricular CMs	Ang II	*failing CMs:*	[48]
			↑ diastolic [Ca^2+^]_i_	
			↓ CaT	
			↓ SR Ca^2+^ load	
			*healthy CMs:*	
			↑ diastolic [Ca^2+^]_i_	
			↑ CaT	
		caged-IP_3_	*failing CMs:*	
			↓ CaT (in failing CMs)	
			↑ diastolic [Ca^2+^]_i_	
			*healthy CMs:*	
			↑ diastolic [Ca^2+^]_i_	
			↑ CaT	
		tetracaine + IP_3_	↑ frequency of Ca^2+^ puffs in healthy and failing CMs	
mouse	atrial CMs	ET-1	↑ frequency of Ca^2+^ sparks	[37]
			↑ SR Ca^2+^ leak	
		caged-IP_3_	↑ frequency of Ca^2+^ sparks	
mouse (IP_3_ overexpression)	atrial CMs	ET-1	↑ frequency of Ca^2+^ sparks	[40]
		PE	↑ occurrence of Ca^2+^ waves	

IICR subsequent to GPCR stimulation is reported to modulate Ca^2+^ signalling during ECC either by directly mediating Ca^2+^ release from the SR or through increasing diastolic [Ca^2+^]_i_, thereby facilitating SR Ca^2+^ release through RyRs [33,35]. By tracking Ca^2+^ responses at individual dyads with a genetically-encoded Ca^2+^ indicator targeted to these sites, we recently demonstrated in paced rat ventricular cardiomyocytes that IICR underlies an increased recruitment of dyads and enhanced SR Ca^2+^ flux at them following ET-1 stimulation [42]. While this effect can be beneficial contributing to acceleration of the Ca^2+^ transient and robust contraction [42], the sensitization of RyRs by IICR can increase the propensity for spontaneous Ca^2+^ releases and the potential for arrhythmogenic Ca^2+^ signals. Indeed, the most consistent effects of IICR observed in ventricular cardiomyocytes across species, including in humans, are induction of arrhythmogenic Ca^2+^ release [30,31,33,60–62] and increased occurrence of Ca^2+^ sparks [28,33,35]. Furthermore, eventless InsP_3_R-dependent Ca^2+^ release that reduces SR Ca^2+^ content (via contribution to SR leak) during ET-1 stimulation is also described in ventricular cardiomyocytes of mice [28]. This InsP_3_-dependent reduction in SR Ca^2+^ content likely results in diminished contraction but is also proposed to protect against arrhythmias [28].

InsP_3_R2 expression is often elevated in ventricular cardiomyocytes of hearts undergoing hypertrophic remodeling or that are in heart failure subsequent to pathological stressors, such as those associated with myocardial infarction or pressure overload [31,45,48,63–66]. The increase in InsP_3_R2 expression parallels that of the re-activated fetal gene programme that is associated with and used as an index of hypertrophic remodeling. Indeed, InsP_3_R2 expression is higher in neonatal hearts and is downregulated with adult maturation [22]. During disease, this increase in InsP_3_R expression has been shown to be mediated by the transcription factor NFATc1 [67] and via post-transcriptional regulation by the hypertrophy-associated microRNAs (miRNA) (e.g. miR-133 regulation of InsP_3_R2 and miRNA-26a of InsP_3_R1 in ventricular and atrial cardiomyocytes respectively) [46,64]. Exacerbating the effect of increased InsP_3_R expression in disease and contributing to an increased function, circulating and local levels of neurohormones and expression of their cardiomyocyte cognate receptors are also upregulated with pathology. As a consequence, the impact of InsP_3_Rs on ECC becomes more important during disease [31,64,65]. Particular effects observed include increased amplitude of systolic Ca^2+^ transients, elevated diastolic Ca^2+^ levels, more frequent arrhythmic events, remodeling of resting membrane potential and prolonged duration of the AP [31,33,65]. Notably, the sufficiency of increased InsP_3_R expression for these effects is demonstrated by the augmented Ca^2+^ release and arrhythmogenic activity observed in InsP_3_R2 overexpressing transgenic mice [28,29]. Augmented InsP_3_ signalling and its generation of elevated diastolic Ca^2+^ levels is also proposed to contribute to the rhythm disturbances and conduction defects in Chagas disease patients (a disease caused by the parasite *Trypansoma cruzi* endemic to latin American countries) [68]. While it is not clear whether InsP_3_R expression is altered in cardiomyocytes from these patients, levels of InsP_3_ are elevated. Inappropriate InsP_3_R signalling leading to Ca^2+^ elevations that do not track the AP-stimulated electrical depolarization of the cardiomyocyte is not in itself sufficient to induce arrhythmias or alter cardiac function. For these ectopic Ca^2+^ elevations to have a wider pro-arrhythmic effect, the Ca^2+^ signal must induce a cellular depolarization sufficient to generate an AP that propagates to neighbouring cells. In this regard, interaction between IICR and NCX has been described in which the increase in intracellular Ca^2+^ generated following InsP_3_R engagement leads to enhanced forward mode NCX activity, thereby augmenting Na^+^ entry into the cell and a slow membrane depolarization that increases the propensity for arrhythmic events [31]. Further supporting this notion, InsP_3_Rs are reported to localize proximally to NCX-enriched domains in the sarcolemma [69].

The almost complete absence or presence of fewer and smaller elementary Ca^2+^ release events in cardiomyocytes in which RyRs are inhibited with tetracaine supports the limited activity of InsP_3_Rs as well as their lower capacity to generate Ca^2+^ signals [28,33,35]. Furthermore, Ca^2+^ release via RyRs was necessary for the full activation of InsP_3_Rs [40]. Since InsP_3_R-mediated elementary events (Ca^2+^ puffs) arise from clusters of two or more InsP_3_Rs channels [70], the poorly detectable nature of IICR could suggest that InsP_3_Rs are not appropriately organized in clusters and are diffusely spread across the SR. This however does not appear to be the case. The suppression of the effects of InsP_3_R activation by inhibition of RyRs has led to the proposal of a mechanism whereby Ca^2+^ release via InsP_3_Rs facilitates RyR opening and enhances their recruitment to generate Ca^2+^ sparks [28,33,35,42]. Further supporting this conclusion, Ca^2+^ puffs that contribute to increased frequency of Ca^2+^ sparks or that trigger RyR opening have been reported [33,40]. The augmentation of RyR-mediated sparks by IICR would require InsP_3_Rs to lie immediately adjacent to RyR clusters [33]. Supporting this hypothesis, we recently demonstrated the presence of approximately 30% and approximately 50% fraction of InsP_3_Rs in the dyad and overlapping with RyRs, respectively, thereby enabling Ca^2+^ signals through InsP_3_R to influence Ca^2+^ release via RyR clusters [42]. At the dyads, Ca^2+^ release via InsP_3_Rs facilitated RyR activation. This action of IICR is likely mediated in two ways - either by direct activation (by CICR) of immediately adjacent RyRs or through increasing dyadic Ca^2+^ thereby bringing RyR in this microdomain closer to threshold for activation, which could then be exploited by stochastically opening RyRs to fully engage the cluster and/or to prevent its detrimental shutdown. In disease, this enhanced IICR-RyR crosstalk may serve to rescue the diminished coupling between LTCC and RyRs and the disrupted Ca^2+^ release due to TT atrophy.

How InsP_3_Rs are targeted to the dyadic region is not fully resolved but the loss of targeting in Ankyrin-deficient mice would suggest that this protein may be involved [69]. The observed selective increase in dyadic InsP_3_Rs relative to nuclear InsP_3_Rs would also suggest that targeting of these two InsP_3_Rs populations is independently regulated [33]. Alternatively, nuclear InsP_3_R expression is invariable and maintained at this location via a separate anchoring protein. InsP_3_Rs were also suggested to lie on regions of the SR devoid of RyRs, perhaps akin to the rogue RyRs proposed to contribute to sparkless leak [71]. This population of InsP_3_Rs has been proposed to elicit its effect via regulation of NCX and/or membrane potential [31].

## 4. Inositol 1,4,5-trisphosphate receptors and nuclear Ca^2+^ regulation

In both atrial and ventricular cardiomyocytes, InsP_3_ signalling potently affects nuclear Ca^2+^ levels [32,33]. Indeed, increases in the amplitude and rate of rise as well as a prolongation of the decay phase of the Ca^2+^ transient is reported in response to stimulation with GPCR agonists or cell-permeant forms of InsP_3_ [32,39,72]. In the absence of Ca^2+^ transients, InsP_3_ promotes nuclear-localized Ca^2+^ elevations, manifested as increased frequency of nuclear and perinuclear Ca^2+^ sparks [39,53]. An elevation in basal levels of nuclear Ca^2+^ also contributes to increased Ca^2+^-dependent gene expression underlying cardiomyocyte hypertrophy [56].

The relatively potent effect of InsP_3_ on Ca^2+^ changes in the nuclear region is likely owing to the greater enrichment of InsP_3_Rs in this region. The nucleus is bounded by the nuclear envelope that serves to separate the genome from the processes in bulk cytosol. The nuclear envelope is densely populated with nuclear pores that permeate entry of proteins less than 30 kDa, ATP and ions including Ca^2+^ to the nucleus. Owing to these properties, the nuclear envelope is not generally considered a barrier to Ca^2+^. As a consequence, cardiomyocyte nuclei are flooded with Ca^2+^ during every Ca^2+^ transient. The nuclear envelope is contiguous with the SR that together form the Ca^2+^ storage compartment of the cardiomyocyte [73]. Furthermore, the nuclear envelope forms invaginations (termed the nucleoplasmic reticulum) that penetrates deep into the nucleoplasm and function as a Ca^2+^ store capable of releasing and removing Ca^2+^ owing to Ca^2+^ release channels and sarco-endoplasmic reticulum Ca^2+^-ATPase (SERCA) pumps that are localized to it, respectively [56,74]. Owing to these properties of the nuclear envelope, nuclear Ca^2+^ signals are subject to regulation by both cytoplasmic Ca^2+^ and local Ca^2+^ signals originating from RyRs and InsP_3_Rs [75,76].

**Figure 3.**
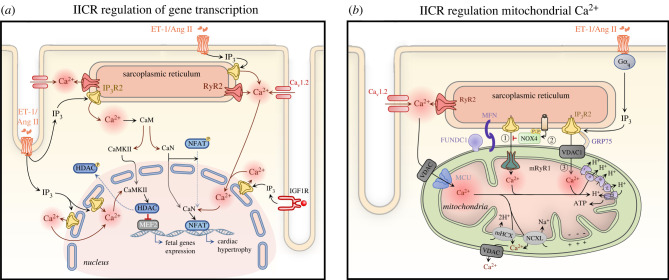
InsP_3_-mediated signalling in regulation of gene expression and mitochondrial function. (*a*) Nuclear and cytosolic Ca^2+^ increases generated by Ca^2+^ release from InsP_3_Rs regulate gene expression underlying cardiomyocyte hypertrophic remodelling. Ca^2+^ released from InsP_3_Rs binds to calmodulin (CaM), which then activates CaM-dependent kinase II (CaMKII) and calcineurin (CaN). Activated CaMKII phosphorylates the inhibitory factor histone deacetylase (HDAC) and induces its export from the nucleus, resulting in MEF2 de-repression and induction of hypertrophic gene expression. Meanwhile, CaN dephosphorylates the nuclear factor of activated T-cells (NFAT) promoting its nuclear translocation and hypertrophic gene transcription. Ang II, angiotensin II; CaM, calmodulin; CaMKII, Ca^2+^/calmodulin-dependent protein kinase II; Ca_v_1.2, *α*1C, subunit of voltage-gated L-type calcium channel; CaN - calcineurin; ET-1, endothelin-1; HDAC, histone deacetylase; IGF1R, insulin-like growth factor 1 receptor; IP_3_, inositol 1,4,5-trisphosphate; IP_3_R2, inositol trisphosphate receptor type 2; MEF2, myocyte enhancer factor-2; NFAT, nuclear factor of activated T cells, RyR2, ryanodine receptor type 2. (*b*) Mitochondrial Ca^2+^ uptake sites are closely localized to Ca^2+^ release sites at the junctional SR forming ‘hotspots’ with the help of tethers MFN and FUNDC1. Ca^2+^ released from the SR via RyRs is taken up via the voltage-gated anion channel (VDAC) associated with the mitochondrial Ca^2+^ uniporter (MCU) (1). In mitochondria, Ca^2+^ controls ATP production and apoptosis. Ca^2+^ is extruded from the mitochondria through Na^+^/Li^+^/Ca^2+^ exchanger (NCXL) and Ca^2+^/H^+^ exchanger (mHCX). Upon stimulation of G*α*_q_ by ET-1, Ang II or NE, InsP_3_ activates Ca^2+^ release from the SR leading to its uptake into the mitochondrial matrix through either VDAC (2) or mRyR1 (4). While Ca^2+^ transfer via VDAC1—GRP75—InsP_3_R results in induction of cell apoptosis (2), when taken up through mRyR1 it is associated with increased ATP production (4). To counterbalance InsP_3_-mediated mitochondrial Ca^2+^ overload during cellular stress, NOX4 augments the level of active phosphorylated AKT, which in turn phosphorylates and suppresses InsP_3_Rs thereby inhibiting Ca^2+^ flux from the SR to mitochondria (3). Akt, protein kinase B; Ang II, angiotensin II; ET-1, endothelin-1; FUNDC1, FUN14 domain-containing protein 1; GRP75, chaperone 75 kDa glucose-regulated protein; MCU, mitochondrial Ca^2+^ uniporter; MFN, mitofusin; mHCX, mitochondrial Ca^2+^/H^+^ exchanger; NCXL, Na^+^/Li^+^/Ca^2+^ exchanger; NE, norepinephrine; NOX4, NADPH oxidase 4; VDAC, voltage-gated anion channel. (Online version in colour.)

Functional and structural evidence indicate that InsP_3_Rs can mediate the generation of nuclear Ca^2+^ signals independently of the Ca^2+^ transients arising owing to ECC [58]. The subcellular localization of InsP_3_R is a crucial determinants of the generation of highly localized Ca^2+^ signals that modulate the activity of Ca^2+^-dependent transcription factors and regulators, which govern the expression of genes underlying hypertrophic remodelling [39,53,57,77,78] (figure 3). While InsP_3_ was considered a highly diffusible messenger, recent data suggests otherwise, thus raising the importance of proximity of the site of InsP_3_ generation with its target receptor [79]. In this regard, GPCRs are found to reside on the nuclear membrane and on TTs that penetrate the cytosol close to the nuclear envelope [80–82]. Additionally, InsP_3_ produced downstream of GPCRs located on the plasma membrane can also diffuse to the nucleus and activate InsP_3_Rs [11].

InsP_3_Rs are located on both the inner and outer membrane of the nuclear envelope, as well as in the perinuclear region and on the nucleoplasmic reticulum [38,56,83]. While the expression of InsP_3_Rs on the inner membrane of the nuclear envelope has been debated for some time, the presence of functional InsP_3_Rs facing towards the nucleoplasm is now widely accepted. Indeed, using an elegant approach in which a fluorescent immobile Ca^2+^ buffer was entrapped in the nucleoplasm or in the cytosol, *Zima et al.* demonstrated InsP_3_-dependent Ca^2+^ release into the nucleoplasm [58]. Further supporting this finding, electron microscopy studies show InsP_3_Rs localized to the inner leaflet of the nuclear envelope [56]. InsP_3_Rs on the outer surface of the nuclear envelope release Ca^2+^ into the cytosol that through diffusion may enter the nucleus via nuclear pores [53]. Owing to the lower buffering capacity of the nucleus, diffusion is anisotropic resulting in a greater influence of this Ca^2+^ release on free Ca^2+^ levels in the nucleus than in the cytosol [75,84]. Together, these data clearly support the capacity for InsP_3_R activation to induce alterations in nuclear Ca^2+^ signalling.

Nucleoplasmic Ca^2+^ homeostasis is altered in cardiac hypertrophy and failure and is thus considered to be associated with the underlying transcriptional changes. During disease, nuclear volume is increased and the density of nuclear invaginations decreased contributing to elevated nuclear [Ca^2+^] at diastole and slower kinetics of nuclear Ca^2+^ transients [46,56]. These effects may in part be owing to the lower surface to volume ratio of the nucleoplasmic reticulum to nucleoplasm, which would reduce the effectiveness of Ca^2+^ clearance mechanisms. Changes in the expression levels of perinuclear InsP_3_Rs, RyRs, SERCA and proteins of the nuclear pore complex are also reported, thereby influencing nuclear Ca^2+^ dynamics [56]. Significantly, upregulation of InsP_3_Rs (particularly type 1 and 2) appears to be central to the aforementioned changes in nuclear Ca^2+^ handling and subsequent transcriptional regulation [46,54,56,85,86].

## 5. Inositol 1,4,5-trisphosphate receptors in transcriptional regulation during cardiac remodelling

Increases in InsP_3_R expression, as well as the activity of mechanisms responsible for InsP_3_ generation, are observed in hypertrophy, heart failure and atrial fibrillation.

In addition to influencing ECC, IICR also has a signalling function in the heart, which is involved in stimulating hypertrophic remodelling of cardiomyocytes. The significance of this mechanism is further reinforced by the observation that expression of InsP_3_R and associated Ca^2+^ fluxes are upregulated in different animal models of cardiac hypertrophy and in human heart failure. Indeed, in human and animal models of heart failure and atrial fibrillation (AF), the increased expression of InsP_3_R2 (heart failure) and InsP_3_R1 (AF) in the nuclear/perinuclear regions was observed and associated elevated nuclear resting Ca^2+^ levels has been assumed to enhance activity of transcriptional factors that regulate pro-hypertrophic gene expression [46,56]. Given its dominant expression in both atria and the ventricle and the effects observed in gain and loss of function studies, a role for the InsP_3_R2 in promoting hypertrophic remodelling is supported [29,39,50,56,64]. While less reported, a role in hypertrophy induction for all three InsP_3_R isoforms in hypertrophic remodelling has been proposed [17]. For instance, InsP_3_R1 is invoked in driving atrial remodeling during AF [46]. Interestingly, overexpression of the InsP_3_R2 in the heart was shown to be sufficient for inducing cardiac hypertrophy in transgenic mice that can be further exacerbated by isoproterenol infusion (β-adrenergic stimulation) and exercise stimulation [29]. The activation of nuclear InsP_3_-induced Ca^2+^ signalling followed by induction of hypertrophy is also well-established *in vitro* in response to autocrine/paracrine neuroendocrine factors (ET-1, Ang II, catecholamines and ATP) acting via G*α*_q/11_ and IGF-1 induced G*α*_i­_-PLC-InsP_3_ signalling [39,77,81,87]. While gating of the tetrameric InsP_3_R requires binding of InsP_3_ and Ca^2+^ [7], the further responsiveness of this channel is regulated by post-transcriptional modifications and via association with regulatory proteins [43,88,89], which may also determine, as in the case for the K-Ras associated protein, whether the receptor is susceptible or licensed for activation [90]. In cardiomyocytes, neuronal calcium sensor-1 associates with InsP_3_Rs enhancing Ca^2+^ release, leading to the triggering of cardiac hypertrophy through engagement of both CaMKII and calcineurin (CaN) pathways [91]. Chromogranin B, which is a Ca^2+^ binding protein forming a complex with InsP_3_R, is also detected in cardiomyocytes, where its expression is upregulated during Ang II-induced hypertrophy [92]. Upon Ca^2+^ binding chromogranin B modifies the magnitude and velocity of IICR and promotes fetal gene expression via the transcription factor nuclear factor *κ*B [92]. InsP_3_R2 gating can also be negatively regulated by CaMKII-mediated phosphorylation, thereby providing a feedback mechanism for InsP_3_R activation [43].

While many studies show a key role of nuclear InsP_3_R in inducing hypertrophy, the contribution of cytosolically-located InsP_3_Rs to this process remains to be fully resolved. Ca^2+^ signals promote gene expression changes required for hypertrophy via modulation of both nuclear and cytosolic Ca^2+^ which lead to the activation of Ca^2+^-dependent transcriptional regulatory pathways, including the CaN/nuclear factor of activated T cells (NFAT) and CaMKII/histone deacetylase 4 and 5 (HDAC)/myocyte enhance factor 2 (MEF2) signalling pathway [39,46,77,87]. Although these pathways can be engaged by global changes in Ca^2+^, this mechanism would not allow the cell to discriminate between changes in Ca^2+^ involved in contraction, which are enhanced during periods of stress. To ensure only appropriate activation of hypertrophic gene expression, a number of mechanisms have been proposed that allow selective encoding of hypertrophy. These include, alterations in the frequency, amplitude, duration (the duty cycle) and location of the Ca^2+^ signal. By modifying Ca^2+^ transients associated with ECC and by having the capacity to influence nuclear Ca^2+^ in a selective manner, IICR may contribute to regulation of transcription in several ways, which may be required in toto for the maximal effects to be manifest. In support of a mechanism involving the generation of spatially localized and regulated nuclear Ca^2+^ signalling microdomains independent of cytosolic Ca^2+^ release, nuclear-specific expression of either Ca^2+^-buffering proteins or InsP_3_ chelators abrogate hypertrophic remodelling in isolated cardiomyocytes [39,72]. Further, enhancement of the magnitude of global Ca^2+^ signals is not sufficient to induce hypertrophy [14,39,46,72,77]. Using a computational modelling approach, Hunt *et al.* proposed that InsP_3_R activation in the cytosol drives NFAT nuclear translocation via modulation of the global Ca^2+^ transient in a way that extends the time when Ca^2+^ levels are above the threshold required for NFAT activation [93]. Active CaN dephosphorylates and complexes with NFAT in the cytosol, although cardiomyocytes also express CaN in the nucleus [54]. The CaN-NFAT complex then translocates to the nucleus [94,95]. Elevated nuclear Ca^2+^ levels subsequently maintain the integrity of the CaN-NFAT complex necessary for sustained NFAT dephosphorylation and nuclear residence required for its full transcriptional activity [94,95]. While activation of CaN appears to be InsP_3_-dependent, the precise mechanism is not yet known [87]. CaN engagement is however activated by sustained local elevations in resting [Ca^2+^]_i_ [54,96]. In cardiomyocytes, this Ca^2+^ signal can be produced either via InsP_3_-mediated increased Ca^2+^ leak from the SR [37,42] or via induction of store-operated Ca^2+^ entry [97]. During hypertrophy and heart failure, elevated Ca^2+^ levels in the nucleus generated via InsP_3_R2 has been consistently shown, thereby providing a mechanism for sustained CaN/NFAT signalling independent from cytosolic Ca^2+^ [39,54,56].

Ca^2+^ release through InsP_3­_R2 can induce hypertrophic gene expression additionally via activation of CaMKII*δ*B in the nucleus [77]. In mediating atrial remodelling associated with atrial fibrillation, Ca^2+^ signals arising from InsP_3_R1 also engages CaMKII. CaMKII regulates transcriptional processes via phosphorylation of transcription factors (e.g. MEF2, CREB, Nkx2–5, GATA4, etc*.*) and epigenetic regulators and histone deacetylases (e.g. HDAC4, 5, 7 and 9) [98]. Of particular importance to the regulation of cardiac gene expression, CaMKII phosphorylates class II HDACs leading to de-repression of gene expression mediated by the hypertrophy-related transcription factor MEF2 [99]. To bring about this effect, phosphorylation of HDACs by nuclear CaMKII*δ*B induces association with 14-3-3 proteins, resulting in nuclear export of the complex, while phosphorylation by cytosolic CaMKII*δ*C blocks HDAC nuclear import [77,100]. To date, the Ca^2+^ source leading to activation of cytosolic CaMKII*δ*C in the cardiomyocytes is not fully known. However, a recent study by Qi *et al.* demonstrated that autophosphorylation of CaMKII*δ*C was prevented by knockdown of *ITPR1* [46].

While cues for physiological and pathological hypertrophy are considered to engage distinct pathways leading to different outcomes, InsP_3_-mediated Ca^2+^ signalling has also been shown to be required for the hypertrophic response to IGF-1 stimulation [72]. The downstream effectors of IICR generated downstream of IGF-1-induced PLC activation are however largely unknown. IGF-1 stimulation was shown to activate MEF2C in an InsP_3_- and nuclear Ca^2+^-dependent manner [101]. Whether this effect is mediated via activation of CaMKII-HDAC or CaN-NFAT pathways remains to be elucidated.

## 6. Role of inositol 1,4,5-trisphosphate receptors in the regulation of mitochondrial Ca^2+^

ECC has a high energetic cost consuming the largest part of ATP produced in cardiomyocytes [3]. The ATP required for cardiac contraction is primarily generated by oxidative phosphorylation in the mitochondria [102,103]. While fatty acids are the major substrate for ATP generation by cardiomyocytes, other sources of energy (e.g. glucose, ketones, amino acids) are also used when available [104]. These alternate sources are particularly used in the ageing or failing heart to compensate for the metabolic insufficiency and loss of ATP generation owing to mitochondrial dysfunction [105,106]. In engaging these alternate energy sources, Ca^2+^ signalling from InsP_3_Rs mediates increased cellular glucose uptake in response to insulin stimulation through GLUT4 translocation and fusion with the sarcolemma [107].

Mitochondria accumulate Ca^2+^ via the voltage-dependent anion channel (VDAC) on the outer mitochondrial membrane [108] and the mitochondrial uniporter located on the inner mitochondrial membrane in a Ca^2+^-regulated manner [109]. Increased mitochondrial Ca^2+^ in turn stimulates Ca^2+^-dependent dehydrogenases involved in oxidative phosphorylation [110,111] (figure 3b(1)). Through this mechanism, intracellular Ca^2+^ levels are coupled with mitochondrial metabolism. For example, under conditions of increased cytosolic Ca^2+^ fluxes (exercise, *β*-adrenergic stimulation), mitochondrial Ca^2+^ accumulation is enhanced, thereby boosting ATP production to provide for the increased demands of ATP consuming pumps [112]. Under pathological conditions, over-accumulation of Ca^2+^ within mitochondria leads to activation of programmed cell death pathways and increased oxidative stress [113]. Ca^2+^ overload brings about this effect through activating the mitochondrial chaperone cyclophilin D (CypD) that induces opening of permeability transition pore (mPTP) [114]. Mitochondrial Ca^2+^ uptake also contributes to shaping cytosolic Ca^2+^ dynamics [115,116].

Ca^2+^ uptake by mitochondria occurs at membrane contact sites known as mitochondrial-associated membranes (MAMs) [117]. In line with other cell types and tissues, SR-mitochondrial Ca^2+^ flux involving InsP_3_Rs at MAMs is supported and regulated by association with the VDAC1 and the mitochondrial stress 70 protein (chaperone 75 kDa glucose-regulated protein; GRP75) in a macromolecular complex [114,118] (figure 3b(2)). In this context, the InsP_3_R1 is invoked and together with VDAC1 are the channels involved in Ca^2+^ transfer between SR and mitochondria respectively, while GRP75 links both channels through binding to their cytosolically facing regions. The importance of this pathway in the induction of mitochondrial permeability during stress is underlined by the increased interaction between CypD and the InsP_3_R-GRP75-VDAC1 complex under conditions of greater mitochondrial Ca^2+^ content [114]. In line with the widely reported involvement of InsP_3_Rs in endoplasmic reticulum (ER)-mitochondrial signalling and cell death induction, augmented InsP_3_-mediated Ca^2+^ fluxes to the mitochondria also have a pro-apoptotic effect in ischaemia-reperfusion (IR) injury [119,120]. During IR injury, this increase in InsP_3_R-mitochondria transfer is brought about by glycogen synthase kinase 3*β* (GSK3*β*) mediated phosphorylation of InsP_3_R1 [120]. Linking stress and mitochondrial Ca^2+^ homeostasis and metabolism, mitochondrial Epac1 complex interacts with and promotes InsP_3_R-GRP75-VDAC1 complex formation under IR conditions, leading to mitochondrial Ca^2+^ overload and opening of the mPTP [121]. First described in a cancer cell context [122–124], phosphorylation of InsP_3_Rs by the protein kinase Akt has also been shown to suppress ER-mitochondrial Ca^2+^ transfer and cell death in the heart. Following IR injury, the abundance of Nox4 at MAMs is increased where it, through generation of reactive oxygen species activates Akt, which in turn phosphorylates and inhibits InsP_3_Rs [119] (figure 3b(3)). Whether and in what context anti-apoptotic members of the Bcl-2 family of proteins interact with and suppress Ca^2+^ transfer via InsP_3_Rs to the mitochondria in cardiomyocytes, as they do in other cell types, remains to be fully explored [125]. By contrast, Seidlmayer *et al.* reported that Ca^2+^ released from the SR via InsP_3_Rs, activated downstream of ET-1 stimulation, is taken up by mitochondria via mitochondrial type 1 RyR (mRyR1) resulting in increased ATP generation in both quiescent and electrically stimulated cells (figure 3b(4)) [126].

Potentially contributing to the diverging effects of InsP_3_ signalling on mitochondrial function in the heart is the presence of different mitochondrial populations that have distinct roles in cardiac physiology. Particularly, interfibrillar mitochondria, which provide ATP for contraction, subsarcolemmal mitochondria, which provide ATP for active transport processes across the sarcolemma, and perinuclear mitochondria, which generate ATP necessary for nuclear processes [127]. Remarkably, Ca^2+^ uptake by interfibrillar and perinuclear mitochondria following InsP_3_-mediated Ca^2+^ release is twice that taken up by subsarcolemmal mitochondria [127].

Further influencing local Ca^2+^ delivery to the mitochondria are electron dense physical linkages called tethers. In cardiomyocytes, these tethers comprise either mitofusin 2 (MFN2) [127] or FUN14 domain-containing protein 1 (FUNDC1) [128]. Ablation of either MFN2 or FUNDC1 was shown to disrupt the association between mitochondria and ER/SR, impair Ca^2+^ uptake into mitochondria and consequently suppress mitochondrial respiration and apoptosis. While both MFN2 and FUNDC1 regulate InsP_3_R-mitochondria crosstalk by maintaining the integrity of MAMs, FUNDC1 was also shown to directly interact with InsP_3_R2 and regulate its stability [128]. Remarkably, FUNDC1 expression is reduced in hearts from patients with dilated cardiomyopathy and in mice with acute myocardial infarction [128].

## 7. Inositol 1,4,5-trisphosphate receptors in cardiac rhythm and conduction

In addition to their role in contractile atrial and ventricular cardiomyocytes, InsP_3_Rs are also functionally expressed in sino-atrial node (SAN) and Purkinje cells. In these cell types, InsP_3_R activation contributes to rhythm regulation and AP propagation, which given the influence of these cells on contraction of the myocardium has significant consequences for cardiac function. In SAN and Purkinje cells, InsP_3_Rs are relatively more abundant than in atrial and ventricular cardiomyocytes. As shown for atrial and ventricular cardiomyocytes, InsP_3_R activation in both of these cell types is amplified by Ca^2+^ release via RyRs leading to more substantial consequences for cellular Ca^2+^ signalling [129,130]. In SAN cells, spontaneous SR Ca^2+^ release events underlie a Ca^2+^ clock involved in pacemaker activity [131]. By stimulating the activity of the electrogenic NCX and membrane potential, this intracellular Ca^2+^ clock interacts with a membrane clock centred on the funny current (I_f_) conveyed by hyperpolarization-activated cyclic nucleotide-gated (HCN) channels [131,132]. Ca^2+^ release via InsP_3_R2 increases the frequency of the spontaneous SR Ca^2+^ release events and Ca^2+^ waves, which by stimulating NCX activity, accelerates membrane depolarization resulting in the threshold for AP generation being more rapidly reached [129,133]. This potent effect of IICR is lost in mice deficient in InsP_3_R2 and under conditions of InsP_3_R inhibition [133]. Further, experiments in knockout mice revealed that augmentation of spontaneous Ca^2+^ wave frequency (the Ca^2+^ clock) by IICR was independent of NCX, thereby demonstrating the importance of IICR in heart rhythm regulation [129]. Through this mechanism IICR contributes to the actions of α-adrenergic, as well as other relevant GPCR agonists [44,129,133]. In addition to this mechanism for regulation of pacemaker activity by IICR, a more recent study from Terrar and colleagues proposed a mechanism whereby IICR elicits its effects via cAMP generated through stimulation of proximally located Ca^2+^-sensitive adenylate cyclases (AC1 and 8) [44]. The cAMP generated acts in turn either directly on HCN channels that underlie the funny current (I_f_), or via PKA and its modulation of the Ca^2+^ handling machinery [44]. The localization of InsP_3_Rs to sub-sarcolemmal regions of the SR in the vicinity of sarcolemmal HCN channels makes this mechanism possible [129,133]. In contrast to the mouse studies of Ju *et al.* and Kapoor *et al.,* baseline pacemaking activity was not influenced by loss of IICR in the murine atrial preparations used by Capel *et al*. The reason for this discrepancy is not clear but could be owing to preparation (cells versus tissue), or the species studied. In Purkinje cells in which the InsP_3_R1 is predominant [130,134], InsP_3_R activation is proposed to contribute to the generation of pathological arrhythmias [135]. This mechanism has been reported in Purkinje cells that survive in the infarcted heart [135]. In these cells, through engagement of RyRs, Ca^2+^ release via InsP_3_Rs leads to the generation of Ca^2+^ waves emanating from the nuclear and sub-sarcolemmal regions of the cell where InsP_3_R expression is enriched [130]. Through engaging NCX, these Ca^2+^ waves may then give rise to triggered activity [136].

## 8. Concluding remarks and future challenges

The diversity of functions that Ca^2+^ controls in the cardiomyocyte raises a significant problem for its ability to concurrently and specifically regulate them. Key to the overlapping and non-overlapping functions of InsP_3_ signalling in the heart is the localization of InsP_3_Rs to cellular Ca^2+^ microdomains coupled with the involved effectors — the dyad with RyRs, the nucleus with associated transcription factors and their regulators, and at MAMs with mitochondria and the Ca^2+^ uptake machinery. The capacity for cardiomyocytes to function in the absence of InsP_3_Rs despite their involvement in multiple cardiomyocyte functions is not clear. Compensation for InsP_3_R2 by upregulation of other InsP_3_R isoforms is one possibility. This potential redundancy is however overcome by use of genetically encoded inhibitors of InsP_3_ signalling such as of the InsP_3_ 5-phosphatase or of a high affinity version of the ligand binding domain of InsP_3_R1 (InsP_3_ sponge) [137], which has the added advantage that it may be targeted to subcellular domains of interest. Both the InsP_3_ sponge and the InsP_3_ 5-phosphatase have been applied to analysis of hypertrophic signalling, and in the case of the InsP_3_ sponge, proven to be effective in *in vivo* studies [29,39]. Whether cardiomyocytes tolerate long term expression of these constructs necessary to test the lifelong role of InsP_3_ signalling, including through cardiac development has not however been determined. To allow acute analysis of the role of InsP_3_Rs in cardiomyocyte physiology without the cell culture required for adenoviral-mediated expression of InsP_3_ probes or interfering RNAs or for transgenesis, improved drugs that target the InsP_3_R are required. For inhibition of InsP_3_Rs, a toolbox including Heparin [138], Xestospongins B, C and D [139], derived from the marine sponge *Xestospongia exigua* and 2-aminoethoxydiphenyl borate (2-APB) is relied upon [140,141]. While heparin is an effective antagonist of the InsP_3_R [138], it is not cell permeant and has effects on RyRs, GPCR coupling and the InsP_3_ 3-kinase [142]. Xestospongins and 2-APB are however cell permeant and can thus be employed in intact cells and tissues. However, these agents have several drawbacks, which should be considered when interpreting studies in which they are used. Xestospongin C for example, in addition to its reported antagonism of the InsP_3_R, equally suppresses SERCA pump activity leading to Ca^2+^ store depletion [142]. This effect of Xestospongins can lead to a misinterpretation of data showing a loss of the Ca^2+^ mobilizing activity of InsP_3_ or of an InsP_3_ generating agonist in experiments in which it is applied. Specifically, IICR may be lost owing to store depletion rather than InsP_3_R inhibition. Xestospongin B was reported to elicit a more selective effect on InsP_3_R without the off target effects on SERCA [139]. Xestospongin D also lacks effects on SERCA but sensitises Ca^2+^ release via RyR [143]. In a more recent study, in experiments designed to directly examine IICR release [138], no inhibitory effect of Xestospongin C or Xestospongin D on InsP_3_Rs was detected, thereby further supporting an indirect mechanism of action for these agents on IICR. Of the InsP_3_R inhibitors used in cardiomyocytes, 2-APB is most reliable and widely used. Like the aforementioned inhibitors, off target effects of 2-APB on store-operated Ca^2+^ entry, mitochondria and SERCA pumps have however been reported. Careful titration of 2-APB in cardiomyocytes, showed that when applied at a low concentration of ∼2 µM, selective inhibition of InsP_3_Rs is achieved with no effects on Ca^2+^ transients or SR store loading detected [30,33,144]. Caffeine also inhibits InsP_3_Rs but owing to its potent activation of RyRs has limited use in cardiomyocytes [138]. As a complement to experiments involving InsP_3_R inhibition, InsP_3_R may also be activated pharmacologically. To these ends, cell permeant forms of InsP_3_ or caged derivatives are employed in intact cells and InsP_3_ salts and caged derivatives introduced via patch pipettes [33,36,37]. The oxidizing agent thimerosol has also been used in cardiomyocytes to induce InsP_3_R activation [31]. While this mercury based agent may sensitise InsP_3_Rs, it has multiple other targets including induction of Zn release from cellular stores [145]. The issues raised above highlight the need for future development and application of improved InsP_3_R probes such as those involving modified versions of InsP_3_ [146] or of the carbon ring on which it is based [147]. These new tools should be used together with advanced imaging approaches to selectively interrogate the localization and function of InsP_3_Rs. Development of strategies to selectively modulate InsP_3_Rs in distinct cellular microdomains and to relocalize InsP_3_Rs to different cellular microdomains to selectively influence discrete functions may also prove of use — for example to prevent arrhythmogenic Ca^2+^ signals. Moreover, application of these approaches in large preclinical models of cardiac disease as well as in human cardiomyocytes is necessary to fill our gap in knowledge regarding the role of InsP_3_R signalling in human pathology.

## Data Availability

We have analysed previously published data. The data are freely available in the previous publication.
